# Measuring Thiols in Single Cultivar South African Red Wines Using 4,4-Dithiodipyridine (DTDP) Derivatization and Ultraperformance Convergence Chromatography-Tandem Mass Spectrometry

**DOI:** 10.3390/foods7090138

**Published:** 2018-08-30

**Authors:** Mpho Mafata, Maria A. Stander, Baptiste Thomachot, Astrid Buica

**Affiliations:** 1 Department of Viticulture and Oenology, Stellenbosch University, 7600 Stellenbosch, South Africa; mafata@sun.ac.za (M.M.); thomachot.b@gmail.com (B.T.); 2 Central Analytical Facility, Stellenbosch University, 7600 Stellenbosch, South Africa; lcms@sun.ac.za

**Keywords:** thiols, wine, derivatization, DTDP, convergence chromatography, supercritical fluid chromatography (SFC), UPC^2^-MS/MS, SPE

## Abstract

Wine varietal thiols are important contributors to wine aroma. The chemical nature of thiols makes them difficult to measure due to low concentrations, high sensitivity to oxidation, and low ionization. Methods for the measurement of thiols usually consist of multiple steps of sample preparation followed by instrumental measurement. Studies have collected large datasets of thiols in white wine but not in red wine, due to the lack of availability of suitable methods. In this study, for the first time, convergence chromatography was used to measure thiols in red wine at ultratrace levels with improved sensitivity compared to previous methods. Performance parameters (selectivity, linearity, limits of detection, precision, accuracy) were tested to demonstrate the suitability of the method for the proposed application. Red wine thiols were measured in South African Pinotage, Shiraz, and Cabernet Sauvignon wines (*n* = 16 each). Cultivar differentiation using the thiol profile was demonstrated.

## 1. Introduction

The volatile fraction of wine is arguably the most important contributor to wine aroma and flavor. Thiol compounds derived from grape precursors, fermentation, and postfermentation/aging treatments provide some important and desirable flavor attributes to both red and white wines [1,2]. 3-Mercapto-1-hexanol (3-MH), 3-mercaptohexyl acetate (3-MHA), and 4-mercapto-4-methylpentan-2-one (4-MMP) contribute fruity aromas to white wine, but their measurement in red wine and contribution to red wine aroma have been little investigated [2,3]. 4-MMP is associated with “black currant” in French red blends [2] and furanmethanethiol (FMT) with “coffee” descriptors [4,5].

Measurement of these wine thiols commonly includes an extensive sample preparation step where the thiols can be extracted free [6], bound using a mercury-based chelator [7,8], or chemically modified using a derivatizing agent [1,9,10]. In order to eliminate matrix interference, the extraction can be liquid–liquid or solid phase, requiring from 20 to 180 mL of wine sample. The extracts are concentrated as part of the extraction step and/or before instrumental analysis, and may further require a solvent switch to allow instrumental compatibility. Due to the long and laborious sample preparation, the number of samples that can be analyzed in one batch is limited. Additionally, internal standards must be used to address accuracy and repeatability issues. Deuterated compounds are preferred for the internal standard, especially since mass spectrometry (MS) is the detection of choice, but deuterated thiols are not commercially available.

The sample preparation is followed by instrumental separation, which commonly consists of gas chromatography (GC) of free or derivatized forms [1,4,8,11], or liquid chromatography (LC) of derivatized forms [6,10]. This is the stage that presents the least issues in the process.

As previously mentioned, MS is the detection of choice for thiol analysis. Thiols intrinsically have low ionization potential and therefore their signal in MS is weak. There are various ways to address this issue. Concentration during sample preparation can increase the levels up to 1000-fold (for example, from 180 mL to approximately 200 µL [6]). Derivatization results in the formation of a thiol derivative that is more stable than the corresponding free form, helping with chromatographic separation and boosting the MS signal due to improved ionization efficiency. Most recently, tandem MS (MS/MS) has been used to increase sensitivity [6,10].

A recent method using 4,4-dithiodipyridine (DTDP) derivatization of thiols tackled some of the previous limitations by decreasing the volume of sample needed and reducing sample preparation time due to the feasibility of derivatization of thiols at wine pH [10]. Furthermore, this MS/MS method offered lower limits of detection compared to previous methods.

In the current study, the instrumental part of the method based on DTDP derivatization was optimized and tested for convergence chromatography (CC). CC is an updated version of supercritical fluid chromatography (SFC) and uses supercritical CO_2_ as the mobile phase. Supercritical CO_2_ is miscible with a wide range of both polar and nonpolar solvents. This makes it more versatile for method development compared to LC and GC. The list of compatible solvents is vast, and in most instances the need to switch solvents before injection is unnecessary, as CC allows for direct injection of most organic solvents. The CO_2_ mobile phase is also compatible with a wide range of stationary phases. The efficiency of CC lies in the supercritical nature of CO_2_ as a low-viscosity mobile phase (high diffusion), which results in better peak resolution and shorter run times compared to LC. At the moment, there is no report in the literature on the use of CC to determine thiols in wine, even though the technique is appropriate with regard to sensitivity of analysis. As an application, thiols (3-MH, 3-MHA, 4-MMP, and FTM) were measured in South African single cultivar Shiraz, Pinotage, and Cabernet Sauvignon wines. This application was chosen based on the scarcity of information on the thiol composition of single cultivar red wines.

## 2. Materials and Methods

### 2.1. Chemicals and Materials

All prepared solutions are expressed in terms of volume percent (%, *v*/*v*), with the balance composed of Milli-Q water, unless otherwise specified. All reagents—3-MH, 6-mercapto-1-hexanol (6-MH), 3-MHA, 4-MMP, FMT, 98% DTDP, ethylenediaminetetraacetic acid disodium salt (EDTA–Na_2_), methanol, 96% ethanol, sodium hydroxide (NaOH), tartaric acid, anhydrous acetaldehyde ≥98%, and 37% hydrochloric acid (HCl)—were purchased from Sigma-Aldrich (Louisville, MO, USA) and the solid phase extraction (SPE) cartridges (Supelclean ENVI-18 SPE) from Supelco (Bellefonte, PA, USA ).

### 2.2. Sample Preparation

**Figure 1:**
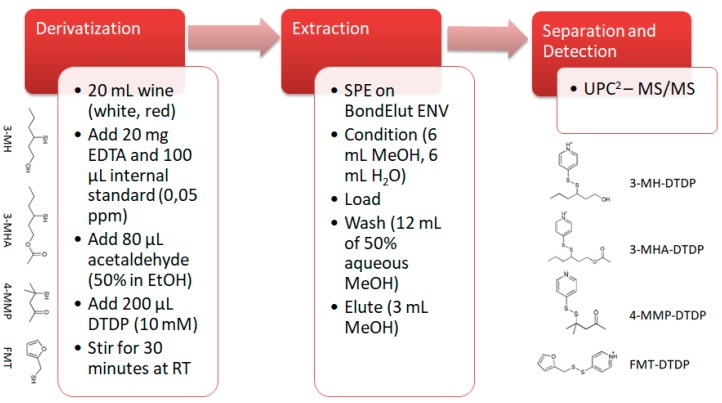
Sample preparation and instrumental analysis of varietal thiols in white and red wine through 4,4-dithiodipyridine (DTDP) derivatization. EDTA, ethylenediaminetetraacetic acid disodium salt; SPE, solid phase extraction; RT, room temperature; UPC^2^, ultraperformance convergence chromatography; MS/MS, tandem mass spectrometry; 3-MH: 3-mercaptohexanol; 3-MHA: 3-mercaptohexyl acetate; 4-MMP: 4-mercapto-4-methylpentane-2-one; FMT; furanmethanethiol; the DTDP appendix designates the respective derivative.

The sample preparation was based on the method developed by Capone et al. [10] with some modifications. To 20 mL of wine, 100 µL of a 0.05 mg/L ethanoic solution of the internal standard (IS, 6-MH) was added. This was followed by the addition of 20 mg of EDTA-Na_2_, 80 μL of 50% acetaldehyde (in ethanol) and 200 μL of aqueous DTDP (10 mM). The mixture was stirred at 500 rpm for 30 min at room temperature. The SPE cartridge was conditioned with 6 mL of methanol followed by 6 mL of water. The sample was loaded onto the cartridge and washed with 12 mL of 50% methanol and dried under vacuum for 5 min. The derivatives were eluted with 3 mL of methanol and injected directly without further concentration. A schematic of the analysis is shown in Figure 1.

### 2.3. Instrumentation and Conditions

Quantitative analysis was performed on a Waters Acquity Ultraperformance Convergence Chromatography (UPC^2^) device using a Waters Viridis BEH 2EP Column (130 Å, 1.7 µm, 3 mm × 100 mm; Waters, Milford, MA, USA). The column temperature and automated back-pressure regulator (ABPR) were set to 60 °C and 2000 psi, respectively. Solvents were CO_2_ and methanol, with a total flow rate of 1.5 mL/min, and the gradient is shown in Table 1. The injection volume was set at 1 µL. The total run time was 7 min including the equilibration step.

Quantitative mass spectrometry detection was carried out using a Xevo TQ-S triple quadrupole mass spectrometer (Waters, Milford, MA, USA). A makeup pump was attached to the coupler that fed 1% (*v*/*v*) formic acid in methanol into the mixer preceding the MS line at a constant flow rate of 0.2 mL/min. Thiol-DTDP derivatives were analyzed in multiple reaction monitoring (MRM) mode using an electrospray probe in the positive ionization mode (ESI+). The following settings were used: capillary voltage 3.8 kV, source temperature 120 °C, desolvation temperature 500 °C, desolvation gas 1000 L/h, and cone gas 150 L/h. MRM settings are shown in Table 2. Data collection and analysis were performed using MassLynx 4.1 (Waters). 

### 2.4. Analytic Method

#### 2.4.1. Selectivity

The selectivity of the chromatographic method was evaluated in model wine, white wine, and red wine. Each thiol-DTDP derivative was injected and the transitions were recorded in multiple reaction monitoring mode (MRM). Two MRM transitions were recorded in addition to the retention times for future peak identification and quantitation, ensuring supplementary selectivity for the method.

#### 2.4.2. Linearity

Linearity was tested by a series of additions of 3-MH (50, 100, 250, 500, 1000, 2000, 2500 ng/L), 3-MHA (25, 50, 125, 250, 500, 1000, 1250 ng/L), 4-MMP (2.5, 5, 12.5, 25, 50, 100, 125 ng/L) and FMT (1, 2, 5, 10, 20, 40, 50 ng/L) to model wine, white wine, and red wine. The model wine was a solution of 12% ethanol in Milli-Q water and 5 g/L tartaric acid, adjusted to pH 3.5 with 10 mM NaOH. All calibrations included blanks (with only IS). Linearity was determined for the 3 matrices based on the regression coefficient (*R*^2^) of the calibration curves. The limits of detection (LOD) and quantification (LOQ) were calculated at signal to noise (S/N) ratios of 3 and 10, respectively, and reported in the 20 mL original sample to allow comparison with the previous study.

#### 2.4.3. Precision and Accuracy

Precision was determined for the entire procedure, from derivatization to instrumental analysis, through repeatability tests. Repeatability (expressed as % relative standard deviation (RSD)) for each matrix was measured in triplicate at 500 ng/L for 3-MH, 250 ng/L for 3-MHA, 25 ng/L for 4-MMP, and 10 ng/L for FMT. Accuracy was also determined at the above-mentioned levels in triplicate through recovery tests (Table 3).

### 2.5. Samples 

Commercial wines were sourced from local supermarkets. Cabernet Sauvignon (*n* = 16), Shiraz (*n* = 16), and Pinotage (*n* = 16) wines were selected to reflect variation in region and vintage. These samples were chosen based on the wine descriptors available at the time of purchase (back labels and tasting notes).

### 2.6. Statistical Analysis

Statistical analyses were performed with SIMCA (version 14.1, MKS Umetrics, Umea, Sweden) and Microsoft Excel 2017 (Microsoft, Redmond, WA, USA).

## 3. Results

### 3.1. Method Performance

Method performance parameters are tabulated in Table 3. The regression for all compounds of interest was linear over the calibration range, with *R*^2^ values above 0.93. The calibration slopes for the compounds of interest in white and red wine were similar to that for model wine, indicating that future calibrations could be done in model wine for unknown samples with minor over- or underestimations. The LODs for 3-MH and 3-MHA were similar to those reported by Capone et al. [10]. The LODs of 4-MMP and FMT were better than previously reported [10]. All LODs for the compounds of interest were lower than their odor thresholds, making this method suitable for combined chemistry and sensory experiments.

### 3.2. Thiol Levels in South African Red Wines

The reported method was used to measure thiols in South African red wines (Table 4). Shiraz and Pinotage wines had broader ranges of thiols than Cabernet Sauvignon. The range of FMT in Pinotage (0.9–186 ng/L) was 5 times wider than Shiraz (0.8–36.3 ng/L) and 18 times wider than Cabernet Sauvignon (0.5–10.2 ng/L). The overall range of 3-MH in all cultivars was broad (min-max 77–363 ng/L, 287 ng/L difference), with Shiraz being the most varied. The overall range of 4-MMP in all cultivars was the narrowest (0.3–3.2 ng/L, 3.2 ng/L difference), followed by 3-MHA (4.7–23.7 ng/L, 19.1 ng/L difference). The average thiol concentrations were above the odor threshold, with the exception of 4-MMP levels in four Pinotage wines (PT5, 12, 13, and 15) and two Shiraz wines (SH2 and 15), and FMT for CS12, which was below the LOD. Average 3-MH and FMT levels in Pinotage (194 ng/L and 58 ng/L, respectively) were higher than Shiraz (169 and 9 ng/L, respectively) and Cabernet Sauvignon (90 and 4.7 ng/L, respectively). Pinotage wines belonging to two wineries (PT11 and 12, and PT4 and 5) had very high FMT levels over the two vintages tested (2016 and 2017). The average 3-MHA and 4-MMP concentrations in Cabernet Sauvignon (23.3 ng/L and 2.8, respectively) were higher than Pinotage (8.3 ng/L and 1.2 ng/L, respectively) and Shiraz (5.8 ng/L and 2.1 ng/L, respectively). Shiraz wines had the lowest thiol levels compared to the other two cultivars.

## 4. Discussion

Considering the selectivity, linearity, precision, and accuracy results, the developed UPC^2^-MS/MS method proved applicable for thiol analysis of both white and red wine. In terms of method performance, the use of convergence chromatography improved the sensitivity of the previous method [10]. Previous methods had to concentrate samples multiple-fold to achieve acceptable signal-to-noise ratios [1,7,10]. For this reason, it can be argued that overall, the current method is more sensitive than the method of Capone et al. [10], even though the reported LODs are similar. The use of supercritical CO_2_ as the main mobile phase in convergence chromatography makes the technique extremely sensitive. When CC is further coupled to a highly sensitive detector such as MS/MS, it results in a very powerful combination capable of analyzing thiols in wine at ultratrace levels (i.e., ng/L). Due to the improved sensitivity of the UPC^2^-MS/MS method, the original concentration step after SPE extraction [10] was excluded, thereby concentrating the sample only 6.67 times (from 20 to 3 mL) instead of 20 times (from 20 to 1 mL). Since the samples were injected directly following SPE, they were less concentrated than in the original DTDP method. In other words, in the current method the sample can be concentrated to a lesser extent and still achieve similar performance in terms of LOD due to the sensitivity of the instrumental technique used. Further concentration can always be included in the sample preparation if a particular application demands it.

Although it is more common to use deuterated compounds, 6-MH was proven to be an acceptable internal standard. The behavior of 3-MH and 3-MHA, compared to 4-MMP and FMT, throughout the sample preparation and detection was better modelled by 6-MH due to their similarities in chemical structure and character. All thiols were, however, modelled well in all wine matrices with acceptable linearity, accuracy, and reproducibility calculated based on 6-MH. The use of a commercially available internal standard is one of the advantages of this method.

The run time per sample was also reduced from 36 min in the previous method [10] to 7 min, making this method less time-consuming.

The wines sampled in this study were selected based on their varied styles, vintages, and tasting notes posted on the back label of the bottles. This study is among only a few reporting thiol concentrations in red wines internationally. The current study presents the first quantitation of FMT in South African red wine. The presence of FMT and its association with coffee aroma in South African Pinotage has previously been shown; the study by Naudé and Rohwer was able to detect FMT by GC-olfactometry but did not quantify it [5].

**Figure 2:**
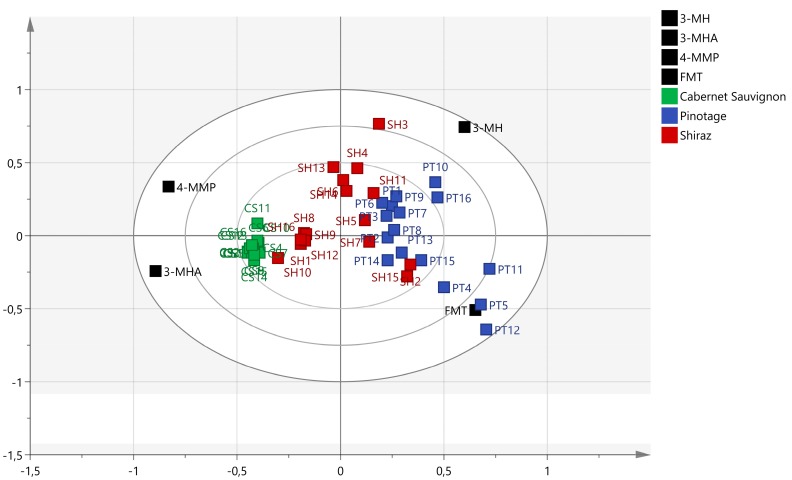
Biplot of thiols in South African Pinotage, Shiraz, and Cabernet Sauvignon (*n* = 16 for each cultivar).

The specific South African cultivars analyzed (Pinotage, Shiraz, and Cabernet Sauvignon) were distinguishable from one another based on their thiol content (Figure 2). Pinotage wines were characterized by high FMT concentrations, Cabernet Sauvignon by a narrow range of concentrations for all thiols, and Shiraz by low average concentrations for all thiols. The narrow ranges in thiol concentrations of Cabernet Sauvignon wines (visualized by the tight grouping in Figure 2) suggests that perhaps the style of wine for this cultivar in South Africa is not very diverse. Shiraz and Pinotage wines, however, had broad concentration ranges for the thiols, which suggests that they have more diverse styles.

The Pinotage wines analyzed were labelled as having “coffee” or “chocolate/mocha” aromas, a particularly sought-after style of Pinotage in South Africa and easily available commercially. These wines were therefore understandably associated with higher FMT levels, since, according to the literature, this thiol is described as imparting a “coffee” aroma [5].

Cabernet Sauvignon wines were associated with higher levels of 4-MMP, which has previously been described as “black currant” [2]. Californian Cabernet Sauvignon wines (*n* = 20) surveyed for 3-MH, 3-MHA, and 4-MMP showed comparable levels in thiols, with the exception of the 3-MH concentrations (396 to 765 ng/L) [13], which were much higher than in the South African Cabernet Sauvignon wines. Other studies that measured red wine thiols did so in other cultivars. Studies in Chilean Carmenère [12] and French appellation red blends [2] reported higher levels of 3-MH (422 to 760 ng/L and 678 to 11,487 ng/L, respectively), with the French blends reporting the highest levels of 4-MMP (5 to 54 ng/L). The Chilean study was the only other study reporting on FMT concentrations, the average of which (34 ng/L) was much higher than the ones for South African Shiraz and Cabernet Sauvignon, but lower than Pinotage.

## 5. Conclusions

The study reports on the first use of convergence chromatography to analyze thiols in wine. The use of convergence chromatography to measure varietal thiols in both white and red wine showed an improvement in sensitivity for the four compounds (3-MH, 3-MHA, 4-MMP, and FMT) measured compared to previous methods. The study shows that the derivatization of wine thiols using DTDP coupled with UPC^2^-MS/MS is fit for the purpose of measuring thiols in white and red wine at ultratrace levels. In addition to extensive thiol composition in single cultivar red wines being reported for the first time, the use of this method revealed thiol-dependent cultivar differentiation.

This type of high-throughput analysis offers faster access to information on the level of thiols in a variety of red wines. Its sensitivity can contribute to the elucidation of the chemical-sensorial role thiols play in red wine through their interactions with one other, with the volatile and nonvolatile matrix, ultimately for better correlation of chemical composition with sensory perception.

## Figures and Tables

**Table 1:** Gradient conditions for UPC^2^-MS/MS analysis of DTDP derivatized thiols.

	Time (min)	Flow (mL/min)	% A (CO_2_)	% B (MeOH)	Gradient Curve
1	Initial	1.5	99	1	
2	2.7	1.5	92	8	5
3	4.5	1.5	90	10	8
4	5.0	1.5	70	30	6
5	5.5	1.5	70	30	6
6	5.7	1.5	99	1	6
7	7.0	1.5	99	1	6

**Table 2:** Mass transitions and analyte retention times for UPC^2^-MS/MS analysis of DTDP derivatized thiols using multiple reaction monitoring (MRM).

Compound	Derivative	Retention Time (min)	MS/MS Transition (*m*/*z*)
3-mercaptohexyl acetate (3-MHA)	3-MHA-DTDP	1.47	286 → 111
			286 → 144
2-furanmethanethiol (FMT)	FMT-DTDP	1.52	224 → 79
			224 → 143
4-mercapto-4-methylpentan-2-one (4-MMP)	4-MMP-DTDP	1.62	242 → 111
			242 → 144
3-mercaptohexanol (3-MH)	3-MH-DTDP	3.08	244 → 111
			244 → 144
6-mercaptohexanol (6-MH, IS)	6-MH-DTDP	3.20	244 → 111
			244 → 144

IS, internal standard.

**Table 3:** Figures of merit for UPC^2^-MS/MS analysis of DTDP derivatized thiols, in model wine (MW), white wine (WW), and red wine (RW).

Compound—Matrix		OT (ng/L)	Calibration Range (ng/L)	*R* ^2^	LOD (ng/L)	LOD * (ng/L)	LOQ (ng/L)	LOQ * (ng/L)	Repeatability ** (RSD%)	Accuracy (%)
**3-MH**	*MW*	60	50–2500	0.9691	3.8	6.4	22.6	21.0	18	101
	*WW*			0.9764	4.0	8.3	24.0	27.5	10	109
	*RW*			0.9911	3.5	10.6	21.2	35.4	9	95
**3-MHA**	*MW*	4.2	25–1250	0.9697	2.3	2.2	13.9	7.4	11	102
	*WW*			0.9821	2.1	1.3	12.4	4.3	11	96
	*RW*			0.9800	3.4	2.2	10.2	7.2	13	119
**4-MMP**	*MW*	0.8	2.5–125	0.9891	0.42	0.8	2.5	2.6	10	89
	*WW*			0.9833	0.20	0.9	1.2	3.1	12	85
	*RW*			0.9832	0.15	1.6	1.8	5.3	8	91
**FMT**	*MW*	0.4	1–50	0.9307	0.13	0.7	0.8	2.3	9	114
	*WW*			0.9711	0.17	1.0	1.0	3.3	11	94
	*RW*			0.9495	0.13	1.5	1.7	5.0	9	101

* Previously reported, using same sample preparation, and LC-MS/MS analysis [10]. ** Of entire procedure, including sample preparation and instrumental measurement. OT, odor threshold; LOD, limit of detection; LOQ, limit of quantification; RSD, relative standard deviation.

**Table 4:** Thiol concentrations in South African Pinotage (PT), Shiraz (SH), and Cabernet Sauvignon (CS) expressed in ng/L.

Pinotage					Cabernet Sauvignon					Shiraz				
ID (Vintage)	3-MH	3-MHA	4-MMP	FMT	ID (Vintage)	3-MH	3-MHA	4-MMP	FMT	ID (Vintage)	3-MH	3-MHA	4-MMP	**FMT**
PT1 (2015)	215	7.6	1.6	11	CS1 (2015)	78	23.2	2.7	1.8	SH1 (2017)	111	<LOD *	2.1	2.9
PT2 (2015)	141	6.9	1.0	3.8	CS2 (2015)	77	23.2	2.8	2.8	SH2 (2016)	110	5.6	0.7 *	33.7
PT3 (2016)	188	7.4	1.5	9.2	CS3 (2016)	81	23.7	2.8	5.3	SH3 (2015)	363	<LOD *	2.7	0.8
PT4 (2016)	155	8.8	1.3	142	CS4 (2016)	93	23.3	2.7	1.0	SH4 (2015)	241	4.7	3.1	9.6
PT5 (2017)	182	12.2	0.4 *	162	CS5 (2017)	82	23.5	2.7	0.5	SH5 (2017)	176	8.4	2.3	36.3
PT6 (2016)	224	9.6	1.7	8.8	CS6 (2016)	107	23.4	2.8	3.9	SH6 (2014)	232	<LOD *	2.5	15.8
PT7 (2017)	214	9.1	1.1	1.5	CS7 (2015)	88	23.1	2.6	7.8	SH7 (2015)	106	5.3	1.4	5.5
PT8 (2016)	164	7.4	0.9	0.9	CS8 (2016)	82	23.2	2.7	6.8	SH8 (2015)	133	<LOD *	2.3	2.9
PT9 (2017)	246	8.7	1.6	12	CS9 (2015)	82	23.3	2.7	5.8	SH9 (2015)	131	<LOD *	2.2	4.3
PT10 (2016)	311	7.9	1.3	26	CS10 (2016)	116	23.8	2.9	10	SH10 (2014)	76	<LOD *	2.3	5.3
PT11 (2016)	226	6.8	0.9	148	CS11 (2015)	147	23.2	3.2	9.9	SH11 (2017)	209	5.6	2.2	8.7
PT12 (2017)	138	10.6	0.4 *	186	CS12 (2014)	86	23.0	2.6	<LOD *	SH12 (2016)	120	<LOD *	2.1	2.2
PT13 (2017)	127	7.3	0.5 *	5	CS13 (2015)	90	23.2	2.8	3.5	SH13 (2015)	265	<LOD *	2.8	1.8
PT14 (2016)	151	9.4	2.4	112	CS14 (2015)	67	23.2	2.6	5.9	SH14 (2015)	246	<LOD *	2.5	6.4
PT15 (2016)	141	6.7	0.7 *	45	CS15 (2016)	77	23.1	2.6	2.9	SH15 (2016)	69	5.1	<LOD *	4.3
PT16 (2014)	287	6.8	1.6	59	CS16 (2016)	94	23.1	2.8	2.0	SH16 (2016)	121	<LOD *	2.3	4.7
Average concentration	194 ± 53	8.3 ± 1.5	1.2 ± 0.5	58 ± 65		90 ± 19	23.3 ± 0.2	2.8 ± 0.1	4.7 ± 2.9		169 ± 79	5.8 ± 1.2	2.1 ± 0.7	9.1 ± 10.4
Range (min–max)	127–311	6.7–12.2	0.3–2.41	0.9–186		77–147	23.0–23.8	2.6–3.2	0.5–10.2		76–363	4.7–8.4	0.03–3.1	0.8–36.3

* Samples with thiol levels below the odor threshold [12]. <LOD = concentration of analyte below the limit of detection. Average concentrations reported as average ± standard deviation.
